# Allergic Sensitization and Psychosomatic Involvement in Outdoor and Indoor Workers: A Preliminary and Explorative Survey of Motorway Toll Collectors and Office Employees

**DOI:** 10.3390/healthcare12141429

**Published:** 2024-07-17

**Authors:** Concetto Mario Giorgianni, Gabriella Martino, Silvia Brunetto, Federica Buta, Trine Lund-Jacobsen, Alessandro Tonacci, Sebastiano Gangemi, Luisa Ricciardi

**Affiliations:** 1 Department of Biomedical Sciences, Dental, Morphological and Functional Investigations, University of Messina, 98124 Messina, Italy; mariogiorgianni@virgilio.it; 2 Department of Clinical and Experimental Medicine, University of Messina, 98124 Messina, Italy; gabriella.martino@unime.it (G.M.); silviabrunetto90@gmail.com (S.B.); federicabuta@gmail.com (F.B.); gangemis@unime.it (S.G.); 3 School and Operative Unit of Allergy and Clinical Immunology, University of Messina, 98124 Messina, Italy; 4 Department of Endocrinology, Centre for Cancer and Organ Diseases, Copenhagen University Hospital, Rigshospitalet, 2100 Copenhagen, Denmark; trine.lund-jacobsen@regionh.dk; 5 Institute of Clinical Physiology, National Research Council of Italy (IFC-CNR), Via G. Moruzzi 1, 56124 Pisa, Italy; alessandro.tonacci@cnr.it

**Keywords:** indoor and outdoor workers, occupational allergic sensitization, psychosomatic disorders

## Abstract

The incidence of respiratory and cutaneous allergic disorders may be variable if we consider work activity. These disorders are reported in the current literature to have a relevant psychosomatic involvement. The aim of the study was to submit a survey on the self-reported occurrence of allergic respiratory and cutaneous diseases in outdoor and indoor workers to verify the onset or exacerbations of such pathologies, encourage surveillance, and suggest the need for further studies. Two groups of workers were compared when exposed and not exposed to air pollutants. An outdoor population of motorway toll collectors (153 workers; M: 58.03 years old, SD: 6.1; and female prevalence of 66.01%) and an indoor group of office employees (59 workers; mean age 54.44 years, SD: 8.50; and male prevalence of 61.02%) entered the study. The results of three multivariate analyses of the obtained data, investigating contributions of external factors, including age, sex, smoking habits, working type, and seniority, on allergic conditions were significant in both workers’ groups. The findings highlighted that age and smoking habits were significant contributors to allergic conditions, both independently and in combination with other factors, such as sex and working type. The data suggest the presence of phenomena related to different etiological domains, as already reported in the literature. Through the collection of these data, it was possible to highlight the need to analyze clinical signs from different perspectives.

## 1. Introduction

Air pollution is defined as any substance in the air that may harm humans, animals, vegetation, or materials [1]. Traffic-related air pollution has been well-documented to be associated with increased risks of airway diseases in the general population [2], and they can also be secondary to environmental, ecological, psychobiological, psychological, and health-related components. In this sense, air pollution has been widely reported in the literature to impact physical, psychological, neuropsychological, and psychosomatic factors [3,4,5].

The two main fields of investigation are air pollution and work activities. Considering the current literature, a need to investigate factors related to air pollution has emerged, as well as working dynamics, such as stress and pressure on workers, the physical and psychological functioning of workers, and, overall, all these factors build a multifaceted framework.

According to Rathee & Singh [6], subjects living in air-polluted areas show greater psychological discomfort, lower well-being, and low health perception. Decreased forced lung capacity, lower maximum voluntary ventilation, and higher respiration rates have been observed in subjects exposed to pollution.

Psychosomatic disorders correlated to medical respiratory conditions have been detected in subjects exposed to air pollution, to the point of requiring an analysis of their involvement when extended to different geographical areas and conditions [7], as well as evaluating significant risk factors for the subjects involved [8,9].

Many studies have linked the development of asthma to urbanization and exposure to pro-oxidative and traffic-related air pollutants [10]. Asthma is a clinical reality that foresees a multifactorial configuration in etiological terms, including psychological dynamics related to affectivity, alexithymia, issues related to the management of emotional experiences, and related to defensive mechanisms used improperly [11,12,13,14,15,16,17,18,19,20].

This attitude occurs not only in subjects with asthma but also in other different chronic conditions, grouped into dermatological [21,22,23,24], gastrointestinal [25,26,27,28,29,30], cardiological [31,32,33,34,35], and respiratory domains other than asthma [36,37,38,39]. Psychological conditions linked to work activities in subjects with chronic diseases have found a wide resonance in the scientific literature [40,41,42,43,44].

In particular, it is known that factors such as burdens, adaptation disorders, and working conditions with relevant environmental dynamics, such as pollution, are risk factors for the onset of various psychopathological conditions [45,46,47,48,49].

From a biological perspective, air pollutants such as nitrogen dioxide (NO_2_), ozone (O_3_), and respirable particulate matter are usually classified by size or aerodynamic diameter (PM2.5-defined particles are <2.5 mm in diameter; PM10-defined particles are <10 mm), differ in their chemical composition, reaction properties, emissions, time of disintegration and ability to diffuse in long or short distances [1]. The worker’s exposure to these agents has been documented to be stressful and a possible etiological factor of cognitive, psychological, and, in a broad sense, neuropsychological effects [9,50,51,52,53].

It has been seen that particulate matter, both fine (FP; ≤2.5 µm in diameter) and ultrafine (UFP; ≤0.18 µm in diameter), emitted by diesel engines in vehicular traffic correlate with an increased incidence of asthma and atopy. The particles of particulate matter promote a Th2-mediated inflammatory response through the activation of cytokines, such as IL-4 and IL-13, which upregulate the production of IgE [10]. These phenomena are known in the literature to cause the onset of many psychosomatic conditions [54,55,56,57,58]. Thus, it is essential to understand their relevance in order to structure diagnostic and intervention paths.

There are not many data relating exposure to pollution to the skin [59]. The common mechanism by which pollution can affect skin physiology is attributable to the induction of oxidative stress and inflammation [60]. Recent contributions considered variables such as inflammation and oxidative stress in different fields, such as psychiatric disorders, psychosomatic and psychobiological conditions, as well as physical conditions, all affecting subjects’ quality of life and health status [61,62,63,64,65,66,67]. Such research requires implementation, considering the results and the difficulties still present in linking chronic skin diseases in workers to psychophysiological mechanisms directly centered on the aforementioned lesion and functional domains.

Nowadays, entire populations of workers are not included in scientific studies that could highlight risks and potentially pathogenic dynamics. In this sense, this study constitutes a preliminary exploratory investigation useful to the emergence of relevant phenomena and the understanding of the effects of biological and psychological variables in subjects involved in working activities, including exposure to pollutants or contact allergens.

Motorway toll collectors are generally exposed to the following working risks: poor ergonomics (posture), sleep disorders (night shifts), respiratory (air pollution), and contact with nickel-containing coins [68]. In our study, these factors were evaluated to assess whether their occupational exposure is a favorable substrate for the onset or exacerbation of allergic pathologies, such as rhinitis, asthma, or allergic contact dermatitis. Office workers are indoor workers, generally exposed to video terminal and poor ergonomics risks, but are not exposed to traffic-related environmental pollutants and were considered as a control group in this study.

## 2. Materials and Methods

During a work surveillance visit, two populations of workers from the same motorway company were studied: one of toll collectors (group 1) and the other of office workers (group 2).

The survey involved 153 toll collectors (group 1) working at booths of a Sicilian motorway in the Strait of Messina area and 59 office workers (group 2) of the same company. Group 1 workers (52 males and 101 females) had an average age of 58.03 years (±6.1) and an average working seniority of 24.03 years (±8.50), while group 2 workers (36 males and 23 females) had an average age of 54.44 years (±8.74) and an average working seniority of 26.01 years (±8.87).

**Figure 1:**
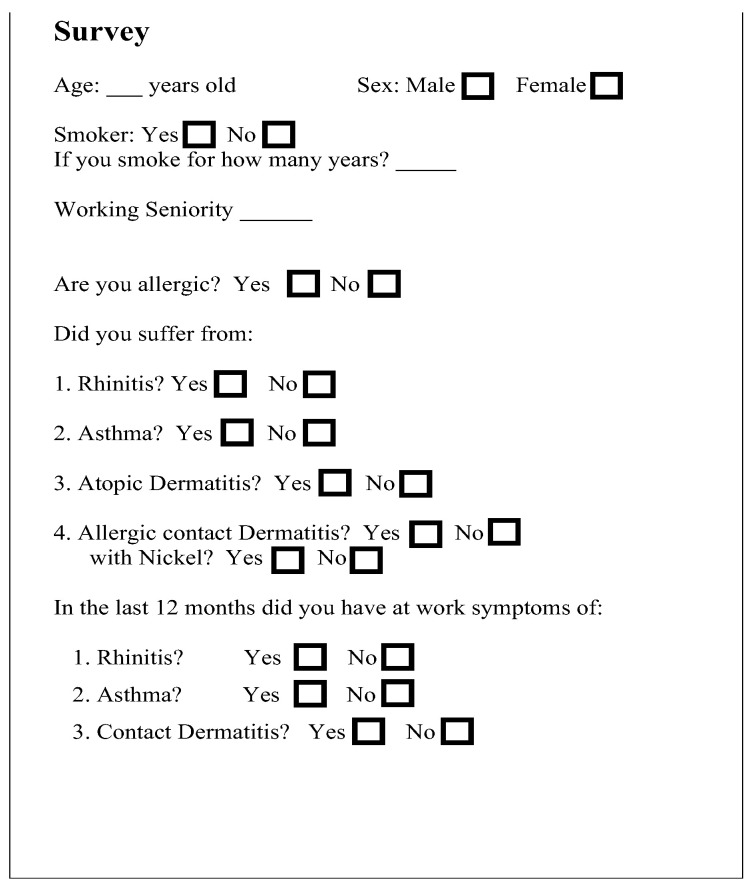
Questionnaire submitted to all workers examined. It consists of four questions about workers’ characteristics, four questions about the history of allergic diseases, and three about the onset of allergic sensitization during the last year.

During a health check carried out by the occupational doctor, they were asked to fill in a survey drawn out by the Operative Unit of Allergology and Clinical Immunology of the University of Messina, answering questions that were reported in the electronic folder available at the G.Martino Hospital in Messina, Italy, was the research was conducted. The questionnaire consisted of 4 questions about patients’ characteristics, 4 about any history of allergic diseases, and 4 about the onset of allergic sensitization during the last year. The questions were explained by the medical staff when submitting the questionnaire to facilitate its comprehension and answering. The questionnaire is reported in Figure 1.

All the workers were informed about privacy and the use of anonymous data for research purposes and signed an informed consent form according to the European GDPR regulation 2016/679. The study was approved by the Ethics Committee of the University Hospital “G. Martino” of Messina, Italy, registration number 24/20. As reported in Figure 1, data were anonymous and collected without the initials of the workers investigated.

## 3. Statistical Analysis

Statistical analysis and graphs were performed using SPSS for Windows (version 17.0). Data are presented as average ± standard deviations (SDs). A chi-square test (χ2) was also performed to compare the observed frequencies of allergic conditions in the two study groups.

Furthermore, three multivariate analyses were carried out to investigate the possible contributions of external factors, including age, gender, smoking habits, working type, and years, on allergic conditions. To each variable, a numerical form was attributed and then reported in the database used for the statistical analysis (see the attached file in the Supplementary Materials). To carry out this analysis, a multivariate General Linear Model (GLM) was adopted, with age and working years categorized into two groups according to the median values, respectively.

## 4. Results

Data analysis of the 153 outdoor workers and motorway toll collectors showed that 53 (35%) were smokers (19 males and 34 females), and 100 (65%) were non-smokers (33 males and 67 females). Among outdoor workers, 20 (13%) had rhinitis (8 males and 12 females), and of these 8 were smokers (2 males and 6 females) and 12 non-smokers (6 males and 6 females), and 10 (7%) had asthma (3 males and 7 females), of which 5 were smokers (1 male and 4 females) and 5 non-smokers (2 males and 3 females). A total of 18/153 (12%) workers reported respiratory symptoms in the last 12 months; 15 (10%) presented with rhinitis symptoms (7 males and 8 females), and 3 (2%), 1 smoker and 2 non-smokers, experienced the exacerbation of asthma symptoms, which refers to an acute onset of symptoms of a chronic disease such as asthma.

Data also showed that 18 workers (11%) (8 males and 10 females) had dermatitis; 5 (3%) (3 males and 2 females) had atopic dermatitis, 13 (8%) (5 males and 8 females) had allergic contact dermatitis of which 10 (7%) had allergic contact dermatitis with nickel (4 males and 6 females). During the working activity in the last year, nine workers (6%) (4 males and 5 females) reported a new onset of contact dermatitis.

Data analysis of the 59 indoor workers and motorway office workers showed that 27 (46%) were smokers (18 males and 9 females), and 32 (54%) were non-smokers (16 males and 16 females).

In this population, 10 (17%) had a history of rhinitis (4 males and 6 females), and of these, 3 were smokers (2 males and 1 female) and 7 non-smokers, (2 males and 5 females); 7 (12%) had asthma, (2 males and 5 females), of which 2 were smokers, (1 male and 1 female) and 5 were non-smokers, (1 male and 4 females). A total of 10/53 indoor workers reported respiratory symptoms in the last 12 months. Rhinitis symptoms were present in 7 workers (12%) (3 males and 4 females), and 3 (5%) experienced the exacerbation of asthma symptoms (1 male and 2 females), all of which were non-smokers.

Data also showed that 10 (17%) indoor workers had a positive history of dermatitis, including 4 males and 6 females, of which 4 (7%) had atopic dermatitis (1 male and 3 females) and 6 (10%) had allergic contact dermatitis with nickel, (3 males and 3 females). During working activity in the last year, 2 (3%) of these workers (1 male and 1 female) presented with a new onset of contact dermatitis (Table 1).

To determine if there was a significant difference in the occurrence of exacerbations for allergic conditions between the two groups of outdoor and indoor workers with different forms of environmental exposure, we performed a chi-square test. The calculated chi-square value (**χ2 = 2.182**) was less than the critical value (α = 0.05: **7.815**), indicating no significant difference between the two groups of workers (Table 2).

Three multivariate analyses were carried out to investigate the possible contributions of external factors, including age, gender, smoking habits, working type, and years, on atopic conditions.

To carry out this analysis, a multivariate General Linear Model (GLM) was adopted, with age and working years categorized into two groups according to the median values, respectively.

The first analysis was conducted by estimating all output variables together (allergy, asthma, rhinitis, AD, DAC, DAC with nickel, recent rhinitis, recent AD, and asthma relapse). Significant effects were calculated, and for them, a partial η^2^ value was also displayed, describing the proportion of total variability attributable to the respective factor, singularly or a combination of them. Of note, η^2^ between 0.01 and 0.06 indicated a small effect of the variable or combination of variables, where a value between 0.06 and 0.14 indicates a medium effect, whereas a value above 0.14 is related to a large effect.

To this end, according to Pillai’s Trace, it was observed that both age (partial η^2^ = 0.103, *p* = 0.014) and smoking habits (partial η^2^ = 0.092, *p* = 0.030) singularly contribute with a medium effect to the onset of the above mentioned clinical conditions, whereas a significant medium contribution was also seen for the combination of gender × type of work (indoor or outdoor) × age (partial η^2^ = 0.101, *p* = 0.015) and for the combination of gender × smoking habits × type of work (indoor or outdoor) × age (partial η^2^ = 0.138, *p* = 0.001).

Secondly, we aimed to estimate the contributing factors to the clinical conditions above-mentioned, yet not recently occurring, therefore eliminating from the output the recent occurrence of rhinitis, AD, and asthma relapse. The results obtained showed a significant medium contribution for age (partial η^2^ = 0.080, *p* = 0.013) and the combination between factors of age × smoking (partial η^2^ = 0.067, *p* = 0.037), gender × type of work (partial η^2^ = 0.064, *p* = 0.048) and, more significantly, for the combination of gender × smoking habits × type of work × age (partial η^2^ = 0.125, *p* < 0.001).

Lastly, we analyzed the recent conditions (recent rhinitis and AD, asthma relapse) as outputs of the model, and to this end, we observed a significant medium effect for smoking (partial η^2^ = 0.061, *p* = 0.007) when considered alone or, as a smaller effect, in combination with the type of work (partial η^2^ = 0.052, *p* = 0.016) (Table 3).

## 5. Discussion

The present study evaluates through a survey the possible onset or exacerbations of allergic respiratory and cutaneous pathologies in motorway toll collectors and office workers from the same motorway company. According to several studies [8,69,70], a strong psychosomatic involvement of chronic conditions, such as allergic respiratory and cutaneous pathologies, can be related to multifactorial etiology. In particular, Tzvian and colleagues [9] highlighted the role of long-term outdoor air pollution exposure, suggesting the need for studies simultaneously investigating air pollution in association with mental health.

It is known that gases and particles present in polluted air can cause damage to both the upper and lower respiratory tracts and induce the onset of clinical conditions such as rhinitis and asthma [71,72]. Both conditions, which can also be present in comorbidity, represent two functional phenomena influenced by different types of variables [73], including affective factors, such as anxiety and depression, and the personality and stress of different orders [74,75,76,77].

People with asthma generally suffer significant pollution effects compared to non-asthmatics [78].

Considering biological factors, the genetic substrate does not fully explain the mechanisms underlying the development of this clinical picture: epigenetics has attracted the attention of researchers by suggesting mechanisms that could lead to a better understanding of the pathogenetic mechanisms [79]. The first contact between atmospheric pollutants and the respiratory system occurs at the level of the epithelial cells, which are in line with the airways and act as an immunological and mechanical barrier: these cells are connected by tight junctions, secrete mucus, and express innate immune receptors that are activated in the presence of pollutants, such as O_3_ and SO_2_, causing damage to the epithelium with the activation of Toll-like receptors and induction of oxidative stress [80]. Pollution has also been reported to induce oxidative stress and inflammation based on the combined effects of ambient air pollutants, such as nitrogen dioxide (NO_2_) and FP matter, with a median aerodynamic diameter of less than 2.5 μm [59]. Inflammation and oxidative stress are the mechanisms by which FP matter determines its effects on health, generating oxygen-free radical activity, DNA oxidative damage, mutagenicity, and the stimulation of proinflammatory factors [81]. The altered function of the mitochondria or nicotinamide adenine dinucleotide phosphate-oxidases (NADPHs) and activation of inflammatory cells causes the generation of ROS and reactive nitrogen species [82]. Some studies have focused on the role of oxidative stress compared to psychosomatic and general psychological variables; in particular, identifying a link between oxidative stress and psychopathological variables could be useful to better understand not only the role of some variables on the onset of conditions but also on comorbidities. Some recent contributions [83] considered the relationship between oxidative stress and dynamics, such as mental disorders, brain functioning, stress, and stress-related disorders, including adaptation and post-traumatic stress disorders.

Considering recent contributions to the understanding of psychoneuroimmunological and psychosomatic factors suggested by Kiecolt-Glaser and colleagues [84] as well as by Sahin and colleagues [85] and Sultana et al. [86], it is particularly interesting to understand all the factors competing with the onset, maintenance, and worsening of such pathological conditions. Recent and relevant studies directly correlated oxidative stress, inflammation, and functional and lesional psychosomatic phenomena, indicating a useful multidisciplinary approach to inspire further studies to investigate competing factors, decreasing the quality of life of subjects and their state of health [56,57,87].

Referring directly to the subjects involved in this study, relatively little has been published on these risks to outdoor workers. Most relevant studies have focused on traffic police, service station workers, and highway maintenance, especially in urban areas with high-traffic pollution; these studies suggest an increase in respiratory symptoms and a decrease in spirometry indices in non-smoking workers in these occupations compared to control subjects [2].

The importance of considering the above variables in workers exposed to pollution is crucial, as suggested by several contributions in the literature. The question concerns areas such as productivity and well-being at work [88], the psychological effects of general exposure [89], economic and social factors [52], as well as general psychological perspectives in different workers’ populations and on a social level [53,90,91]. The data in the literature show the need to consider population groups and workers not previously investigated. In this sense, this study represents an exploratory approach to relevant clinical real-life data that need further attention and detail.

Thus, in our study, data collected through a survey submitted to outdoor and indoor workers from a motorway company in Sicily, in the South of Italy, reported a similar prevalence of respiratory allergic symptoms in both populations, respectively, for rhinitis in 13 and 7% and asthma in 10 and 3%. These ranges are also reported in the general population [92,93]. Therefore, occupational exposure to traffic pollutants did not increase the incidence of allergic symptoms in outdoor workers included in our study. It must also be underlined that these percentages were reported even if the workers had been employed for several years and, therefore, had a long working seniority.

The results of the present study have to be considered preliminary data, and therefore, further research is needed.

A confounding variable in the present research could be that motorway workers considered in this study, both outdoor and indoor, worked in the Strait of Messina area, which is characterized by low pollution levels [94].

Considering the information threshold for the ozone setting (O_3_) and the annual average value of nitrogen dioxide (NO_2_) and FP (PM 2.5 and PM10), it emerges that the air quality in our reference area is better when compared to a city like Genoa with similar geographical characteristics: these data were acquired by the Regional Agency for Environmental Protection [95] in the regions of Sicily and Liguria (Table 4).

Allergic contact dermatitis has proven to be a professional problem affecting quality of life and working capacity, although nickel allergies have a genetic predisposition [96] and are known as a condition of high psychosomatic incidence; therefore, they are dependent on several factors worthy of consideration [97,98]. The hands are commonly involved in occupational contact dermatitis [99]. Nickel is an allergen that commonly causes contact dermatitis [100], and the risk of the elicitation of nickel dermatitis, concerning repeated exposure to low levels of this metal in exposed workers, such as motorway toll workers, is described as nickel is used in coins due to its low cost, shiny surface, and corrosion resistance [101].

Nucera et al. [102] evaluated the presence of nickel in Euro coins; twenty-five patients underwent a patch test with 1- and 2-Euro coins, and nineteen of them were positive. Outdoor toll collector workers and indoor office workers in our population reported a new onset of contact dermatitis in 6% and 3% of workers, respectively, overlapping the prevalence of contact allergies in the European general population [103].

Our study, unfortunately, has some limitations, such as the small sample size and the self-reported nature of the data, even if the multivariate analysis showed a statistically significant result, which highlights the importance of considering both individual and combined effects of external factors on allergic conditions in outdoor and indoor workers. Age and smoking habits resulted as significant contributors, both independently and in combination with other factors, such as sex and working type. The present study is a preliminary exploratory report. Further studies should be based on the data that emerged to perform analyses useful to reveal phenomena related to the onset, maintenance, and exacerbation of pathological conditions interfering with quality of life. Surveys like the one used in this study could be easily administered to investigate the presence of allergic respiratory and skin pathologies in workers to verify and possibly report the onset or exacerbations of such pathologies, improving occupational health policies. In all, employees’ well-being could be further investigated in larger samples to obtain appropriate and informative results, analyzing different variables correlated to allergic sensitization, psychosomatic phenomena, and working environments through a complete allergic and psychometric work-up and evaluating work-related environmental quality.

## 6. Conclusions

Allergens and haptens can cause allergic sensitization, inducing the appearance of various allergic diseases that need early diagnosis for timely treatment. Allergic respiratory and cutaneous sensitization are pathological entities that adversely affect the quality of life and workers’ state of health. Moreover, it has been possible to suggest that the etiology, often not fully known, and competing factors represent a multifactorial configuration that needs to be appraised.

Data obtained during the above-reported work surveillance visit of motorway toll collectors and office workers from the same motorway company through self-reported answers to a survey about allergic respiratory and cutaneous symptoms were encouraging in terms of the low incidence of occupational allergic sensitization, while age and smoking habits were significant contributors both independently and in combination with other factors, such as sex and working type. Nevertheless, the results obtained from different workers with different risk factors rule this study as a starting point for a more articulated consideration of phenomena, their causes, and their relationships with other types of factors.

Other surveillance studies should be performed on these and other categories of workers to follow up on the possible onset of allergic diseases in various geographical areas with different levels of air pollution and different levels of psychosomatic involvement.

## Figures and Tables

**Table 1:** The table shows the prevalence of allergic conditions in outdoor and indoor workers from the same motorway company. Data are stratified by age, sex, smoking habits, and working seniority; the occurrence of allergic conditions in the last year is also reported.

External Factors		Outdoor Workers with Allergic Conditions				Outdoor Workers (No. 153)
		Rhinitis (13%)	Asthma (7%)	AD (3%)	DAC (8%)	
Average Age (μ) ± Standard Deviation (σ)		58.4 ± 6.93	60.4 ± 2.59	61.8 ± 2.68	60.5 ± 3.18	58.03 ± 6.1
Sex	Male	8	3	3	5	52
	Female	12	7	2	8	101
Smoking Habits	Smoker	8	5	2	8	53
	No-Smoker	12	5	3	5	100
Working Seniority (μ ± σ)		25.8 ± 7.92	26.5 ± 5.72	30.8 ± 5.63	27.8 ± 6.28	24.03 ± 8.50
**External Factors**		**Outdoor Workers with Allergic Conditions in the Last Year**				
		**Rhinitis (10%)**	**Asthma (2%)**	**Dermatitis** **(6%)**		
Age (μ ± σ)		60.1 ± 4.37	59.7 ± 2.08	59.4 ± 3.32		/
Sex	Male	7	1	4		/
	Female	8	2	5		/
Smoking Habits	Smoker	6	1	4		/
	No-Smoker	9	2	5		/
Working Seniority (μ ± σ)		28.3 ± 6.08	27.3 ± 5.51	29.8 ± 4.35		/
**External Factors**		**Indoor Workers with Allergic Conditions**				**Indoor Workers (No. 59)**
		**RHINITIS (17%)**	**ASTHMA (12%)**	**AD** **(7%)**	**DAC** **(10%)**	
Age (μ ± σ)		51.7 ± 8.58	52 ± 9.18	51.5 ± 13.13	59.5 ± 4.28	54.44 ± 8.74
Sex	Male	4	2	1	3	36
	Female	6	5	3	3	23
Smoking Habits	Smoker	3	2	2	3	27
	No-Smoker	7	5	2	3	32
Working Seniority (μ ± σ)		23.3 ± 7.59	23.6 ± 8.92	21.3 ± 11.47	27.7 ± 6.41	26.01 ±8.87
**External Factors**		**Indoor Workers with Allergic Conditions in the Last Year**				
		**Rhinitis (12%)**	**Asthma** **(5%)**	**Dermatitis** **(3%)**		
Age (μ ± σ)		51.6 ± 9.71	56 ± 2	59.5 ± 6.36		/
Sex	Male	3	1	1		/
	Female	4	2	1		/
Smoking Habits	Smoker	1	1	2		/
	No-Smoker	6	2	/		/
Working Seniority (μ ± σ)		21.9 ± 9.11	26 ± 7.81	28 ± 11.31		/

**Table 2:** The table describes the results of the statistical analysis performed with a chi-square test to compare observed (O) and expected (E) frequencies of allergic conditions among outdoor and indoor workers.

Condition	Outdoor Workers Observed (O)	Indoor Workers Observed (O)	Total Observed (O)	Outdoor Workers Expected (E)	Indoor Workers Expected (E)	χ2 Outdoor Workers	χ2 Indoor Workers	χ2
Rhinitis	15	7	22	15.87	6.13	0.048	0.122	
Asthma	3	3	6	4.33	1.67	0.409	1.100	
Dermatitis	9	2	11	7.94	3.06	0.134	0.368	
No Allergic Symptoms	126	47	173	125.87	47.13	0.001	0.000	
**Totals**	153	59	212			0.592	1.590	2.182

**Table 3:** This table summarizes the results of the three multivariate analyses conducted, providing an overview of the significant factors and their interactions.

Analysis	External Factor	Partial η^2^	*p* -Value
**First Analysis** Output Variables: allergy, asthma, rhinitis, atopic dermatitis (AD), allergic contact dermatitis (ACD), ACD with nickel, recent rhinitis symptoms, recent AD manifestations, and asthma relapse.	Age	0.103	0.014
	Smoking habits	0.092	0.030
	Sex × Type of work (indoor/outdoor) × Age	0.101	0.015
	Sex × Smoking habits × Type of work × Age	0.138	0.001
**Second Analysis** Output Variables: allergy, asthma, rhinitis, atopic dermatitis (AD), allergic contact dermatitis (ACD), and ACD with nickel (excluding conditions in the last year: rhinitis symptoms, AD manifestations, and asthma relapse).	Age	0.080	0.013
	Age × Smoking	0.067	0.037
	Sex × Type of work (indoor/outdoor)	0.064	0.048
	Sex × Smoking habits × Type of work × Age	0.125	<0.001
**Third Analysis** Output Variables: recent rhinitis symptoms, recent AD manifestations, and asthma relapse.	Smoking	0.061	0.007
	Smoking × Type of work (indoor/outdoor)	0.052	0.016

**Table 4:** In this table, data acquired by the Regional Agency for Environmental Protection of Sicily and Liguria regions are reported considering Messina in Sicily and Genoa in Liguria.

Urban Area	NO_2_	O_3_	PM2.5	PM10
Messina (1)	Annual average 20 μg/m^3^	Day of exceedance OLT: 1	Annual average 11 μg/m^3^	Annual average 22 μg/m^3^
Genoa (2)	Annual average 54 μg/m^3^	Day of exceedance OLT: 7	Annual average 13 μg/m^3^	Annual average 23 μg/m^3^

## Data Availability

Data reported in the manuscript will be made available if reasonably requested.

## Supplementary Materials

The following supporting information can be downloaded at https://www.mdpi.com/article/10.3390/healthcare12141429/s1.
